# Meta-Accuracy of Very First Impressions: A Mini Review

**DOI:** 10.3389/fpsyg.2021.736534

**Published:** 2021-10-13

**Authors:** Elena Tsankova, Ergyul Tair

**Affiliations:** Department of Psychology, Bulgarian Academy of Sciences, Institute for Population and Human Studies, Sofia, Bulgaria

**Keywords:** first impressions, unacquainted persons, meta-accuracy, review (article), introduction, single global measure, componential approach

## Abstract

The meta-accuracy of first impressions (i.e., how accurately one understands others’ perception of oneself) can be conceptualized and measured in various ways. In order to reduce conceptual and methodological overwhelm, facilitate understanding of the topic, and stimulate future work in the field, we conducted a brief introductory literature review on the meta-accuracy of first impressions. Following a definitions-and-methodology-focused overview of the historical development of the topic, we present comparative synthesis and analysis of the key conceptualization and measurement methods used to study the meta-accuracy of first impressions. We also summarize the central research themes and types of stimuli that have been studied in relation to the meta-accuracy of first impressions. Finally, we make several suggestions for further research that could be beneficial to the future development and expansion of the field.

## Introduction

*Jane was shaking as she left her new mentor’s office: “She suggested so many changes… She must have thought I am totally incompetent… How am I ever going to get my degree?!”*

*Professor Jones, still at her desk, smiled as she went over her first meeting with Jane: “What a clever girl! Quite anxious, true, but so resourceful – such an interesting research topic… I hope my feedback helps her get the finer details right.”*

### First Impressions

The opening anecdote describes a situation in which a first impression occurs—that of Professor Jones about her new graduate student Jane. First impressions are the inferences we make about someone upon an initial encounter with them. These impressions are formed quickly and spontaneously (Willis and Todorov, 2006) and are long-lasting (e.g., Gunaydin et al., 2017). First impressions are also remarkably influential as they are known to affect and predict situations of high personal (e.g., employment interviews; Harris and Garris, 2008) and societal relevance (e.g., political elections; Olivola and Todorov, 2010). Given the power of first impressions, understanding their various characteristics becomes essential for ensuring successful communication and avoiding misunderstanding.

### Accuracy

One important characteristic of first impressions is their accuracy. Is the first impression of Professor Jones about Jane accurate? Accuracy in interpersonal perception typically refers to the correspondence between the subjective perception of the interaction partners and some more objective (i.e., more stable with respect to time and influences) criterion (e.g., Funder and West, 1993; Brauer and Proyer, 2020). First impressions can be (e.g., Ambady et al., 1999) but are not necessarily accurate (e.g., Rule et al., 2013; see Wood, 2014 for a detailed review on first impressions accuracy). Divergent findings on interpersonal accuracy may to a large extent be explained by differences in conceptualization and measurement approaches. Funder (1995) discusses common problems with defining and measuring interpersonal accuracy and proposes the Realistic Accuracy Model whose aim is to resolve many of these problems. The model also discusses moderators of interpersonal accuracy, such as the qualities of the perceiver, target, trait of interest, and the information involved.

### Meta-Accuracy

In social interactions, there is a significant amount of trying to figure out what others think. The accuracy with which one infers others’ perception of oneself is termed as *meta-accuracy* (Kenny and DePaulo, 1993). In our opening scenario, meta-accuracy relates to the correspondence between what Professor Jones thinks of Jane and what Jane thinks Professor Jones thinks of her.

It is important to note, however, that the matter of meta-accuracy is far more complex than the mere agreement between target and perceiver and instead is the result of the interplay between various factors and moderators (Funder, 1995; Carlson and Elsaadawy, 2021). Things are further complicated by the target’s and judge’s views having both shared and distinctive components, by targets and judges achieving different levels of accuracy for different information types (e.g., self–other knowledge asymmetry or SOKA; Vazire, 2010), and by “blindness” to how one is perceived under certain circumstances (e.g., Gallrein et al., 2013, 2016).

Understanding the meta-accuracy of first impressions is just as important as understanding their accuracy because it, too, has the potential to shape subsequent interactions. What we believe others make of us may to a large extent determine our own behavioral tendencies. Jane, for example, believes that Professor Jones sees her as incompetent and might try to work harder to prove that she is not and/or engage in self-fulfilling-prophecy behaviors and indeed present herself as incompetent.

### Aim

The meta-accuracy of first impressions can be conceptualized and measured in multiple ways. The diversity in definitions and measurement techniques is advantageous as it allows addressing the phenomenon from various angles. However, it may also become overwhelming, confusing, and cause uncertainty and disagreement. We conducted a brief review to facilitate comprehension of current knowledge and planning of future work on the meta-accuracy of first impressions. We outline central conceptualization and measurement traditions, summarize key topics and stimuli studied in relation to the meta-accuracy of first impressions, and we present several further research ideas.

## Materials and Methods

### Literature Search

In September 2020, we conducted a Google Scholar search for articles in English whose text contained the expressions “meta accuracy” and “first impressions,” combined with the Boolean operator AND. The search returned approximately 144 results which we manually reviewed for relevance. We identified additional articles from the text and reference sections of the relevant Google Search results and from the automatic suggestions on some of their journal pages. Through the literature search and the manual filtering, we ended up with 70 articles.

### Inclusion Criteria

The 70 articles underwent further detailed manual scrutiny with respect to several inclusion criteria. First, articles needed to be based on *empirical data* and be *published peer-reviewed journals*. Although, as stated earlier, first impressions generally tend to be exceptionally persistent, they can still be modified following subsequent exposure to new information under the right circumstances (e.g., Gawronski et al., 2010). As we were interested in the meta-accuracy of not yet modified “very first impressions” (term borrowed from Bar et al., 2006, p. 269), we restricted our review to articles that reported data on *at least one previously unacquainted group or time point with no previous acquaintance*. Finally, since the developmental and clinical perspective were not central to our, we only *included articles that studied at least one non-clinical group* and *excluded articles that studied only adolescent samples*. During the peer review process, one reviewer brought to our attention two recent articles on the “liking gap”—an underestimation of interaction partners’ liking for each other. Those papers study the meta-accuracy of first impressions but had evaded our search due to the use of slightly different keywords. Both articles met our selection criteria and were included in the review. Our final selection consisted of 20 articles.

### Synthesis and Analysis

From these 20 articles, we extracted the central ways of conceptualization and measurement of the meta-accuracy of first impressions, as well as the main research topics and stimulus information types studied in relation to meta-accuracy (Table 1). Based on this synthesis, we identified several prominent research themes and stimuli types already figuring in the study of the meta-accuracy of first impressions. Our synthesis also helped spot a couple of research themes intuitively linked to but not yet studied in this context.

**Table 1:** First impressions meta-accuracy measures, central addressed topics, and information type used to form the impressions.

Article	Meta-Accuracy Measure	Topic	Information Type
Boothby et al. (2018)	regressions	liking gap (underestimation of how much interaction partners like each other)	face-to-face interactions and videos
Carlson and DesJardins (2015) Study 2	SRM	narcissism, popularity	face-to-face interactions
Carlson (2016a)	regressions SAM	psychological adjustment	face-to-face interactions
Carlson and N. (2016b) Study 1	SAM	relationship quality	face-to-face interactions
Carlson et al. (2010)	correlations hints of componential approach	idiographic meta-accuracy, calibration of meta-accuracy	face-to-face interactions
Carlson et al. (2011a) Studies 1, 3	correlations (Study 3) regressions (Studies 1, 3)	meta-insight (distinguishing between how one sees oneself and how others see them)	face-to-face interactions
Carlson et al. (2011b) Studies 1, 2	multilevel modeling (Study 1) SRM (Study 2)	narcissism	face-to-face interactions
Carlson et al. (2017)	SAM	interpersonal problems	face-to-face interactions
DePaulo et al. (1987)	CCAM SRM	dyadic and person (general) accuracy	face-to-face interactions
Lu et al. (2018)	difference scores correlations	eagerness, optimistic bias	face-to-face performances and interactions
Malloy and Janowski (1992)	SRM	leadership	face-to-face interactions
Mastroianni et al. (2021)Study 1	regressions	liking gap(underestimation of how much interaction partners like each other)	face-to-face interactions
Pinkham et al. (2019)	difference scores regressions	schizophrenia	face-to-face interactions and videos of face-to-face interactions
Re et al. (2016)	difference scores regressions ANOVA	self-favoring biases, selfies	selfies and experimenter-taken photos
Reno and Kenny (1992)	SRM	self-consciousness, social anxiety	face-to-face interactions
Rom and Conway (2018) Studies 1–3	ANOVA	morality, moral dilemmas	responses to a moral dilemma scenario
Sasson et al. (2018)	difference scores correlations mean comparisons ANOVA	autism	videos
Stopfer et al. (2014)	correlations regressions	personality expression and impression formation in online social networks (OSNs)	thin slices of OSN profiles (pictures, interests, texts)
Tissera et al. (2020)	SAM	social anxiety	face-to-face interactions
Wu and Zheng (2019)	correlations	impression management in online social network sites; status updates	OSN status updates (texts)

No shading = single global measure approach; light gray = componential approach; and dark gray = combination of single global measure and componential approach.

### Conceptualization and Measurement of the Meta-Accuracy of First Impressions

Before summarizing our observations, we briefly review the history and logic of meta-accuracy conceptualization and measurement.

The literature points to two major ways of conceptualizing meta-accuracy, both rooted in interpersonal perception research. One way is to look at meta-accuracy as a stand-alone phenomenon either devoid of or disregarding any potential effects of the perceiver, target, and the measures. Biesanz (2010) refers to this approach as “a single global measure of accuracy” (pp. 854–855). This tradition typically estimates meta-accuracy as either the correlation (and/or regression) or difference between the target’s evaluation of how they believe the perceiver sees them and the perceiver’s actual evaluation of the target. Comparison of means is also used within this tradition. Going back to our opening example, a single global measure approach would simply match (by correlations, regressions, or mean comparisons). Jane’s belief of how her professor sees her with the professor’s actual perception. Any effects on meta-accuracy caused by Jane and the professor, as well as their attributes (e.g., personality and momentary states), would not be taken into account.

The other way of conceptualizing meta-accuracy is to consider the influence of the perceiver, target, and measures. Such conceptualization produces “componential models” which include the effects of the different components in interpersonal perception (Biesanz, 2010). The first model of this type was Lee J. Cronbach’s components of accuracy model (CCAM), published in Cronbach (1955) and focusing on the interaction between target and measure for each perceiver. Kenny and La Voie (1984) published the Social Relations Model (SRM), also based on componential logic but centering around the interaction (and considering effects of the specific relationship) between perceiver and target for each measure. In 2010, Jeremy C. Biesanz combined the CCAM and SRM into the Social Accuracy Model (SAM), which allows looking at perceiver and target effects across measures and traits. Readers will find an excellent review of componential models in Biesanz (2010). It suffices for our purposes to state that the logic of componential modeling is based on estimating the variance explained by each component (and, when applicable, by the relationship between components). In our opening example, a componential model could tell us how much of the observed effect is due to Jane’s perception, the professor’s perception, their unique characteristics, and their very specific situation (i.e., different from how other people see Jane and from how the professor sees other students). Furthermore, componential approaches, as well as studying meta-accuracy for multiple trait profiles instead of for single traits (e.g., Furr, 2008), allow the decomposition of meta-accuracy into *normative* (stereotypical, related to group perception) and *distinctive* (unique, related to the individual). Thus, although they entail some conceptual and computational differences, componential and profile approaches could tell us what aspects of Jane’s meta-accuracy are stereotypical (related to how Jane believes she is seen by a larger group of people and how they actually see her) and what are distinctive (related to how Jane believes she is seen specifically by Professor Jones and how the professor sees Jane).

The choice of measurement method for meta-accuracy is determined by its conceptualization, which in turn is influenced by researchers’ preferences and tradition, the research question, and/or the availability of resources. Componential approaches generally provide a broader view on meta-accuracy but at the same time require complicated designs and computations. Often, to meet their specific research needs and circumstances, researchers opt for an adaptation and/or combination of approaches.

Out of the 20 articles we reviewed, we classified 10 as using primarily a single global measure, seven as using a componential approach, and three as relying on combinations of both. The choice of conceptualization and measure are typically reasonably justified by theory and/or previous research and follow logically from the studied questions. There is clearly a preference for the componential approach and even specific models within the approach among collaborators with established traditions, while researchers new to the field tend to begin with single global measures and later may add componential measures or switch preferences entirely.

### Addressed Themes

The reviewed articles cover a broad range of topics, which could be organized into several major themes. First, following the historical development of the field, a large portion of the research has been dedicated to uncovering proof of the *existence of the meta-accuracy phenomenon* and *identifying its principal characteristics* (DePaulo et al., 1987; Carlson et al., 2010, 2011a) and *biases* (Re et al., 2016; Lu et al., 2018). Second, meta-accuracy has been studied in the context of specific *personality traits* and *ways of social functioning* (Malloy and Janowski, 1992; Reno and Kenny, 1992; Carlson et al., 2011b; Carlson and DesJardins, 2015; Carlson, 2016a,b, 2017; Rom and Conway, 2018; Sasson et al., 2018; Pinkham et al., 2019; Tissera et al., 2020). The third and most recent theme in the study of the meta-accuracy of first impressions, that is still in its early days, is dedicated to studying the phenomenon in the framework of *Internet communication* (Stopfer et al., 2014; Wu and Zheng, 2019).

### Types of Stimulus Information

The majority of reviewed articles studied first impressions formed in the context of face-to-face interactions. A couple of more recent studies also used videos, photographs, or texts. These observations led us to conclude that although traditionally the meta-accuracy of first impressions has been investigated in direct face-to-face interactions, there is a tendency to reflect societal trends by also addressing novel predominant communication means.

## Discussion

### Summary

We reviewed 20 articles investigating the meta-accuracy of first impressions formed without previous acquaintance between/among the interaction partners. We established that based meta-accuracy conceptualization, research could be organized into three categories—(1) work considering the effects of the perceiver, target, and measures (componential approach), (2) work looking at meta-accuracy in isolation from perceiver, target, and measures effects (single global measure approach), and (3) work combining the two approaches. The way viewing meta-accuracy is determined by the specific research question, circumstances, and tradition.

We also extracted topics studied in relation to first impression meta-accuracy and organized them into three central themes—(1) evidence of, characteristics of, and biases in the meta-accuracy of first impressions, (2) personality traits and specifics of social functioning associated with meta-accuracy, and (3) meta-accuracy of first impressions on the Internet. There is not sufficient number of studies addressing each topic to allow the conclusion that there is a preferred method of conceptualization and measurement associated with a particular topic. Our observations do show that narcissism and social anxiety have been studied together with meta-accuracy using componential approaches, but this could also be explained by the tradition and composition of the research teams.

Finally, we observed that although traditionally research of the meta-accuracy of first impressions has relied on face-to-face interactions, new stimuli reflecting the predominant modes of interpersonal communication are also being incorporated.

### Ideas for Further Research

Within the reviewed articles, we found systematic, but a bit limited in scope, investigation of first impression meta-accuracy. This is fully understandable as the field is relatively small and the literature reflects the methodical work around the primary questions of interest for only a couple of research groups. Researchers new to the field do introduce some diversity in the studied topics (e.g., morality; Rom and Conway, 2018).

One intuitive direction for expanding the scope of the field would be to address a *broader range of individual characteristics* that have the potential to affect first impression meta-accuracy. The most obvious candidates would be perceiver personality differences, such as gender, agreeableness, neuroticism, which, as Hall et al. (2016) suggest, could be linked to the accuracy of personality detection. Further logical candidates for expanding individual characteristics range would be social intelligence and Theory of Mind. Given reports of impaired meta-accuracy in autism (Sasson et al., 2018), it is reasonable to suspect positive associations between social competencies and meta-accuracy. If perspective taking abilities are impaired, one might not be able to properly grasp how another might see them upon their very first encounter. To illustrate with our opening anecdote, Jane may indeed be simply anxious, but she may also have suboptimal or impaired social abilities, causing her to misinterpret social cues and not properly understand what Professor Jones thinks of her. As noted by Kenny and Albright (1987), the role of perceiver and target personality has long been of interest to interpersonal perception accuracy research but has not been properly addressed due to research method limitations. In a way, novel models, such as the SAM, do provide the means to study such questions, and the understanding of the meta-accuracy of first impressions would benefit from a systematic investigation on the matter.

Surprisingly, we did not come across any literature directly reporting the study of meta-accuracy of first impressions from a *neurological perspective*. What kind of meta-accuracy-related neural activation could we expect in Jane during and following her meeting with Professor Jones? We would predict the involvement of networks associated with thinking about what others think (e.g., Theory of Mind; Saxe and Kanwisher, 2003) and representation of the self (e.g., Molnar-Szakacs and Arzy, 2009). But what would the temporal dynamics of this activation be? Would there be a (recursive) feedback-based correction mechanism associated with meta-accuracy and what would that involve? Answering these and further neural activation questions would enhance our understanding of meta-accuracy of first impressions and might point toward previously unaddressed issues related to both its conceptualization and measurement. Recent work by Schindler et al. (2021) provides a peak into this direction by demonstrating through the study of event-related potentials following dyadic interactions that feedback incongruent with one’s self-view influences different processing stages of others’ evaluations of oneself.

Finally, we have seen through both the recently addressed themes and types of stimuli used in impression formation that meta-accuracy research has already began incorporating the specifics of Internet communication. Given the ever-increasing presence of the Internet in daily life, we would like to encourage the continuation and expansion of the Internet-related branch of research on meta-accuracy of first impressions. In particular, we believe that some highly specific characteristics of Internet communication, such as synchronicity, deserve attention as they may affect meta-accuracy of first impressions. Would meta-accuracy be affected (and if so, how) by a time lag between the exchanges in the communication process? Some Internet-specific types of information used in the formation of first impressions, such as avatars, for instance, also present research possibilities for meta-accuracy research.

In short, we propose that the understanding of meta-accuracy of first impressions could be enhanced by (1) expanding the range of individual characteristics that are studied in association with it, (2) addressing its neural correlates, and (3) strengthening its investigation in the context of Internet interactions.

### Limitations

Our work followed a strict protocol but also entails a strong qualitative component. This more descriptive than statistical format is explained by the overall small number of peer-reviewed studies addressing the meta-accuracy of first impressions formed without previous acquaintance. With very few scientists studying the topic directly, in many instances, the conceptualization and measurement approaches as well as the studied theme are determined by tradition and the composition of the respective research team. As the field grows and the literature in it expands, it would become feasible to conduct more systematic reviews and possibly meta-analyses.

### Conclusion

With our overview of the conceptualization and measurement approaches, our summary of the central studied themes, and our overview of potential future research directions we hope to make the domain of meta-accuracy of first impressions easier to navigate and appealing to students and interested researchers. With this review, we wish to introduce the topic in a not-too-daunting way, as well as inspire and facilitate further work in the field.

## Author Contributions

All authors contributed to the conception of the review and the establishment of the article inclusion criteria. ETs conducted the literature search, article classification, and wrote the first draft of the manuscript. ETa provided feedback and contributed to revisions of the draft. All authors approved the submitted version.

## Funding

This work, including open access publication fees, was supported by a grant with contract number КП-06-ДБ-3 from the National Scientific Program “Petar Beron. Science and Innovation with Europe” (Petar Beron and NIE) of the Bulgarian National Science Fund at the Ministry of Education of the Republic of Bulgaria.

## Conflict of Interest

The authors declare that the research was conducted in the absence of any commercial or financial relationships that could be construed as a potential conflict of interest.

## Publisher’s Note

All claims expressed in this article are solely those of the authors and do not necessarily represent those of their affiliated organizations, or those of the publisher, the editors and the reviewers. Any product that may be evaluated in this article, or claim that may be made by its manufacturer, is not guaranteed or endorsed by the publisher.
